# Donor Risk Factors Affecting Graft Survival in Pediatric Kidney Transplants: Protocol for a Systematic Review and Meta-Analysis

**DOI:** 10.2196/71620

**Published:** 2026-02-12

**Authors:** Cahyani Gita Ambarsari, Jon Jin Kim, Nur Hasnah Maamor, Izzah Athirah Rosli, Nor Asiah Muhamad

**Affiliations:** 1 School of Medicine University of Nottingham, University Park, Nottingham, NG7 2RD, United Kingdom, 44 (0)115 951 5151, 44 (0)115 951 3666; 2 Department of Child Health, Faculty of Medicine Universitas Indonesia-Cipto Mangunkusumo Hospital, Central Jakarta, Jakarta, Indonesia; 3 Medical Technology Cluster, Indonesian Medical Education and Research Institute, Faculty of Medicine Universitas Indonesia, Central Jakarta, Jakarta, Indonesia; 4 Department of Paediatric Nephrology, Nottingham University Hospitals, Nottingham, United Kingdom; 5 Department of Surgery, University of Cambridge, Cambridge, United Kingdom; 6 Sector for Evidence-based Healthcare, National Institutes of Health, Ministry of Health, Shah Alam, Selangor, Malaysia

**Keywords:** graft failure, human leukocyte antigen, HLA, hypertension, increased-risk donor, kidney transplant, mismatch, pediatric, weight

## Abstract

**Background:**

Pediatric patients with end-stage kidney disease require kidney transplants (KTs) throughout their lifetime. Long-term graft survival is dependent on multiple factors, which are broadly categorized as donor- and recipient-related factors. Advances in transplant care and changes in donor population demographics necessitate an updated analysis on donor risk factors to guide clinical decision-making.

**Objective:**

In this systematic review and meta-analysis, we will focus on the impact of donor factors on graft survival in pediatric KT, excluding transplants from donation after circulatory death as the latter are less common in children.

**Methods:**

This review encompasses studies reporting donor-related risk factors for graft survival in pediatric KT, including age, size, comorbidities, and ethnicity for living and deceased donors, as well as the cause of death and length of hospitalization for deceased donors. The literature search will use the following databases: PubMed, Scopus, Web of Science, Embase, and Cochrane. Two independent reviewers will select studies and assess their quality. Pooled estimates of relevant factors will be computed via a random-effects model using the Stata/BE (version 19) software. Depending on data availability, subgroup analyses will be conducted based on donor type (living vs deceased). The reporting of findings will adhere to the PRISMA (Preferred Reporting Items for Systematic Reviews and Meta-Analyses) guidelines.

**Results:**

The search and screening for the systematic literature review are anticipated to be finished in June 2026. Data extraction, quality appraisal, and subsequent data synthesis will begin in July 2026. The review is expected to be completed by October 2026, and the study results will be published in 2027.

**Conclusions:**

Our review will provide a comprehensive synthesis of the available evidence on kidney donor risk factors impacting graft survival in pediatric KT. The results of this review could provide valuable insights for clinical decisions, policy development, and ongoing efforts to improve outcomes for children with end-stage kidney disease requiring KT.

## Introduction

Kidney disease, including chronic kidney disease and end-stage kidney disease (ESKD), is a major global health issue, with ESKD posing profound challenges to the health and well-being of pediatric patients and their families [1-9]. Among the treatment options for ESKD, kidney transplant (KT) is the most preferred, demonstrating better outcomes with reduced mortality and morbidity [10-13]. Additionally, pediatric KT creates opportunities for improved growth, development, education, and social interaction, leading to an overall enhanced quality of life for young recipients [1]. Pediatric transplant recipients are projected to live over 50 years and, therefore, will require more than one transplant during their lifetime. Therefore, minimizing the need for multiple transplants and maximizing the lifetime of each transplant is crucial. Acknowledging the multiple risk factors, including both medical and psychosocial factors, we aim to examine donor-related factors that affect long-term allograft survival. The impact of immunological risk factors, including human leukocyte antigen (HLA) mismatching [14-16] and sensitization reflected by panel-reactive antibodies [17], along with recipient-related risk factors such as prior kidney replacement therapy modality [18,19] and its duration [20-22], has been previously reviewed.

Prognostication is important to guide clinical decision-making both at the population level, as facilitated by kidney allocation schemes (KASs), and at the patient level, as informed decision-making between the patient and clinical team. When a kidney offer is presented to a pediatric patient, nephrologists and transplant surgeons must rapidly make intricate decisions regarding the organ’s suitability for transplantation. This process entails evaluating various clinical, psychological, and social factors relying on often incomplete information about the donor and existing knowledge about the patient from the organ procurement organization [23,24]. Factors known to influence decisions include donor quality, recipient risk factors, and physicians’ decision-making behaviors [14,18,25-29]. Nonetheless, trying to weigh each factor is difficult. Clinical risk indexes that combine multiple factors have been developed, but they have mainly used adult population data, and their applicability in pediatric patients has not been proven [26,30-32].

The increasing demand for KT among pediatric patients far surpasses the limited supply of organs from deceased donors, highlighting the necessity to expand the donor pool [33,34]. The scarcity of kidney allografts poses a significant obstacle for patients with ESKD seeking transplants, emphasizing the crucial need to expand the donor pool and optimize the use of available allografts [34]. Additionally, the demographics of the donor population have changed, and donors are becoming increasingly older with more comorbidities [26,33]. Therefore, health care professionals need to be able to make careful considerations on accepting donors with risk factors rather than wait for an ideal donor, which might prolong waiting times [26].

This review aims to examine donor risk factors and their impact on graft survival in pediatric patients undergoing KT. By combining recent evidence, it seeks to offer valuable insights for clinical decisions, policy development, and ongoing efforts to improve outcomes for this vulnerable patient group. Our review aims to address the following question: in pediatric KT, what donor risk factors influence allograft survival? To the best of our knowledge, no systematic review or meta-analysis has previously examined this question.

## Methods

This review will be conducted and reported following the PRISMA (Preferred Reporting Items for Systematic Reviews and Meta-Analyses) protocol guidelines. We will also integrate this review with the MOOSE (Meta-Analyses of Observational Studies in Epidemiology) guidelines as the outcome will be a meta-analysis of selected observational studies.

### Eligibility Criteria

We will include all original articles that meet the following criteria:

- Studies involving pediatric patients as KT recipients (aged <18 years at the time of transplant)
- Studies reporting the first kidney-only transplants, defined as the patient’s first transplant and excluding simultaneous liver-kidney or other combined organ transplants, from living donors and deceased donation after brain death
- Studies that investigate donor-related risk factors for graft survival and report graft survival as the primary or secondary outcome
- Peer-reviewed original articles, including randomized controlled trials and observational studies (cohort, case-control, and cross-sectional studies)
- Articles published or accepted for publication beginning from 2000

The exclusion criteria are as follows:

- Studies involving adult recipients in which the pediatric group data cannot be extracted
- Studies involving transplants from deceased donors following circulatory death in which the donation after brain death group data cannot be extracted
- Studies involving multiorgan transplants in which the kidney-only group data cannot be extracted
- Studies involving kidney retransplantations in which the first KT group data cannot be extracted
- Reviews, case studies, commentaries, qualitative studies, or editorials

### Outcome Measures

The following are the outcomes of this review:

- Death-censored graft survival: graft survival, considering only cases in which graft failure occurred due to factors other than patient death. Graft survival is defined as the time from transplant to graft failure. Graft failure is defined as the start of dialysis or retransplantation. Our primary aim is to evaluate 5-year graft survival. However, we will adhere to the availability of the data identified through our search strategy and anticipate that outcomes may be reported at varying time points, including 1-, 5-, and 10-year graft survival.
- All-cause graft survival: combined outcome of graft survival and patient survival.

### Information Sources

#### Electronic Search

We will systematically conduct a comprehensive literature search using various literature databases (MEDLINE [PubMed], Web of Science, Scopus, Embase, and Cochrane Central Register of Controlled Trials (CENTRAL) to identify eligible studies. Secondary searches will be conducted on Google Scholar and other websites, and the reference section of the included studies will also be hand searched for additional relevant studies. We will include both English- and non–English-language studies in this review. For studies published in languages other than English, translation support will be sought to ensure accurate screening, data extraction, and interpretation. This approach minimizes language bias and ensures that potentially relevant evidence is not excluded based on language of publication. The search will be performed from database inception to May 2026.

#### Search Strategy

The proposed search terms will cover 3 key themes. The first theme will be “pediatric kidney transplantation,” including keywords for children and adolescents. The second theme is “risk factor,” including unrelated, living, or tissue donors and donor selection. The third theme is “graft survival.” The exploded versions of the MeSH (Medical Subject Headings) terms for each theme will be included. All 3 search themes will be combined using the Boolean operators “OR” and “AND.” The detailed search terms for each database are presented in Multimedia Appendix 1.

### Study Selection

We will identify and remove duplicates and collate multiple reports. Two review authors will independently screen all the titles and abstracts to examine the potential studies for inclusion and exclude studies that are clearly irrelevant. We will identify the studies and code them as “retrieve” (eligible or potentially eligible or unclear) or “do not retrieve.” We will retrieve the full-text study reports and publications, and the review authors will independently screen the full texts to identify studies for inclusion as well as identify and record reasons for the exclusion of ineligible studies. We will resolve any disagreements through discussion. If no consensus is reached, the other 2 authors will act as arbiters.

We will document the study selection process in sufficient detail to complete a PRISMA flow diagram and construct a table describing the characteristics of the excluded studies [35,36]. In accordance with PRISMA standards, the flow diagram summarizing records identified, screened, excluded, and included will be incorporated into the final review. Any deviations from or amendments to this protocol will be transparently documented, together with their rationale, and reported in the final review. The EndNote reference management software (Clarivate Analytics) will be used to store, organize, and manage all the articles identified from the databases.

### Data Extraction and Management

We will use a standardized data extraction form created in the Microsoft Excel spreadsheet software for study characteristics and outcome data. Two review authors will independently extract outcome data from the included studies. In the “characteristics of the included studies” table, we will note whether outcome data are reported in a usable way. We will resolve any disagreements through consensus or by involving the other 2 authors. We will double-check that data are entered correctly by comparing the data present in the systematic review with the data in the study reports. The following characteristics from the included studies will be extracted:

- Title, authors, study country, region, and publication year
- Methods: study design, source of data, total duration of the study, and method of analysis
- Participants: number of patients, age range, and sex
- Outcome: graft survival
- Exposures or risks: donor age, sex, diabetes, hypertension, smoking, body height, body weight, ethnicity, cause of death, days in hospital, cytomegalovirus, and hepatitis C virus (however, where applicable, we will extract data on other exposure risks or modifiable risk factors, control conditions, and adjustment variables)

### Quality Assessment

Two review authors will independently assess the studies’ quality based on the criteria in the Newcastle-Ottawa Scale (NOS) for observational studies. The NOS is a widely used tool for assessing the quality of nonrandomized studies, including cohort studies and case-control studies. The NOS applies a star system, in which the study is assessed based on three broad perspectives: (1) the selection of the study groups, (2) the comparability of the groups, and (3) the ascertainment of exposure and outcome [37]. The maximum score is 9 points, and the studies can be classified as being of good, fair, or poor quality according to the following standard thresholds:

- Good quality: 3 or 4 stars in the selection domain AND 1 or 2 stars in the comparability domain AND 2 or 3 stars in the outcome or exposure domain
- Fair quality: 2 stars in the selection domain AND 1 or 2 stars in the comparability domain AND 2 or 3 stars in the outcome or exposure domain
- Poor quality: 0 or 1 star in the selection domain OR 0 stars in the comparability domain OR 0 or 1 star in the outcome or exposure domain

To assess the risk of bias in the included intervention studies, we will use a Cochrane Risk of Bias Assessment Tool for Nonrandomized Studies of Interventions [38]. This assessment tool consists of 5 domains of bias (selection bias, confounding bias, performance bias, detection bias, and attrition bias), which will be assessed using a set of signaling questions. Each domain will be rated as having a low, high, or unclear risk of bias, and an overall risk-of-bias rating will be assigned for each study. The use of a Cochrane Risk of Bias Assessment Tool for Nonrandomized Studies of Interventions will allow us to provide a standardized assessment of the quality of evidence from nonrandomized studies of interventions and determine the overall risk of bias across studies.

To assess the risk of bias in randomized controlled trials, we will use version 2 of the Cochrane risk-of-bias tool for randomized trials, which evaluates bias across domains including the randomization process, deviations from intended interventions, missing outcome data, measurement of outcomes, and selection of reported results [38].

### Statistical Analysis

#### Data Analysis and Statistical Analysis

Statistical analyses will be conducted using the Stata software (StataCorp). The main R packages *meta* and *metafor* (R Foundation for Statistical Computing) will be used for meta-analysis. We will calculate the pooled estimates of graft survival using a random-effects model to allow for heterogeneity across studies. Where appropriate, effect estimates will be stratified according to donor type (living vs deceased) to explore potential differences in outcomes.

#### Assessment of Heterogeneity

Assessing heterogeneity is a critical step in conducting a meta-analysis as it allows for an evaluation of the degree of inconsistency or variability among the results of individual studies. For this review, we will evaluate heterogeneity using both the *I*^2^ and *Q* statistics. The *I*^2^ statistic will be used to quantify the impact of heterogeneity, with percentages of 25%, 50%, and 75% representing a low, moderate, and high degree of heterogeneity, respectively [39]. The *Q* test is a statistical test that determines whether there is significant heterogeneity among the studies. The significance level for the *Q* test will be set at .01 in this review [40]. If significant heterogeneity is detected using the *Q* test or *I*^2^ index (>50%), we will explore potential sources of heterogeneity using subgroup analyses and meta-regression. We will also explore the possible causes (eg, differences in study quality, participants, or outcome assessments) and evaluate the studies in terms of their methodological characteristics to determine whether the degree of heterogeneity can be explained by differences in those characteristics and whether a meta-analysis is appropriate.

#### Assessment of Publication Bias

We will create and examine a funnel plot to explore possible small-study biases if we can pool more than 10 studies in a single meta-analysis. The number of studies missing from the funnel plot will be estimated. The effect size after the inputting of these missing studies will be estimated using the trim-and-fill method. The trim-and-fill method is a simple estimation approach proposed by Duval and Tweedie [41] in which the asymmetric outlying part of the funnel is trimmed, the symmetric remainder is then used to estimate the true center of the funnel, and the trimmed studies and their counterparts are then replaced around the center. Other methods to assess publication bias, including the Begg rank correlation and the Egger weighted regression method test, will also be performed [42,43].

### Ethical Considerations

We registered this systematic review with PROSPERO (CRD42024500442). As this review will use published data, ethics approval has been waived.

### Dissemination

The systematic review will focus on donor risk factors in pediatric KT, the results of which will be made publicly available and disseminated through publication in a peer-reviewed journal after completion and presentation at conferences.

## Results

The search and screening for the systematic literature review are anticipated to be finished in June 2026. Data extraction, quality appraisal, and subsequent data synthesis will begin in July 2026. The review is expected to be completed by October 2026, and the study results will be published in 2027.

## Discussion

We will conduct a systematic review on donor risk factors that affect kidney allograft and patient survival in pediatric patients. This is an important clinical question as it influences the decisions of clinicians and families on the choice of donor to accept, for example, whether there is an upper limit of donor age to accept or whether donor comorbidities (eg, diabetes, hypertension, or obesity) translate to poorer outcomes in the recipient.

Since the first successful KT in 1954, surgical transplantation techniques have evolved considerably. New approaches to transplant protocols have been revised over the past 2 decades to overcome organ donor shortage, for example, careful selection of older living donors, donors deceased from cardiac causes, and donors with a history of increased infection risk [44]. Concurrently, the management of infants on dialysis has improved, and the number of transplants in smaller-sized recipients has increased [45-47]. Immunosuppressive treatment regimens have also evolved, such as the introduction of tacrolimus and mycophenolate mofetil in 1994, which replaced azathioprine and cyclosporine [48]. In parallel, clinical factors such as posttransplant proteinuria have emerged as important markers of graft injury, with strong associations with impaired graft survival and adverse outcomes in pediatric recipients [49-52]. Therefore, a contemporary analysis of the factors affecting long-term transplant survival is required. Our emphasis on uniformity in the inclusion criteria and consideration of the evolution of different practices will help ensure sample representativeness. The design, use of standardized study rating instruments, and adherence to systematic review and meta-analysis guidelines further enhance the study’s robustness.

In view of the advances in KT, our review will focus on studies from the year 2000 onward. During this time, the systems for allocating deceased donor KT have also evolved considerably [53]. In the United Kingdom, a national KAS was first introduced in 1989. HLA matching was introduced in 2006, and utility matching using donor and recipient risk indexes was introduced in 2019 [54]. The United States implemented a national sharing and donor service area system with their KAS in 1987. In 2014, a revised US KAS scheme was implemented. Currently, this system uses the Kidney Donor Profile Index to assess donor kidney quality, with pediatric patients given priority [53,55]. Australia and New Zealand’s allocation system, established in 2008 and revised in 2011, emphasizes national prioritization for well-matched grafts using state-based algorithms for local allocations [56,57]. European countries have collaborated since 1967 on a registry of KT candidates to optimize HLA matching and introduced the Eurotransplant Kidney Allocation System in 1996. This Eurotransplant Kidney Allocation System point scoring system considers factors such as HLA match grade, mismatch probability, waiting time, geographic distance, national balance, medical urgency status, and pediatric age [58]. Therefore, there are variations in KASs internationally reflecting local priorities and variations in the way in which pediatric patients are prioritized [59]. Nonetheless, the donor risk indexes, which assess donor quality, have not been shown to be as effective in prognosticating outcomes in pediatric patients [26,30-32].

We hope that a large meta-analysis that combines results worldwide will overcome the problem of small sample sizes in pediatric KT and improve the validity of the analysis. However, there is a need to acknowledge the potential limitations arising from the diverse designs of the included studies. Our review will use subgroup analyses and meta-regression to address high heterogeneity and enhance the overall quality of evidence. Despite potential biases in observational study designs, the use of standardized study rating instruments, compliance with all relevant guidelines for systematic reviews and meta-analyses, and synthesis of evidence from multiple studies can strengthen the validity of our conclusions.

By providing solid and updated reports on donor risk factors in pediatric KT, this review aims to contribute substantially to the refinement of transplant strategies, policy development, and clinical practice in this critical area of pediatric nephrology.

## Supplementary material

10.2196/71620 Multimedia Appendix 1 Proposed search terms.

10.2196/71620 Checklist 1 PRISMA-P checklist.

## Abbreviations

CENTRAL: Cochrane Central Register of Controlled Trials
ESKD: end-stage kidney disease
HLA: human leukocyte antigen
KAS: kidney allocation scheme
KT: kidney transplant
MeSH: Medical Subject Headings
MOOSE: Meta-Analyses of Observational Studies in Epidemiology
NOS: Newcastle-Ottawa Scale
PRISMA: Preferred Reporting Items for Systematic Reviews and Meta-Analyses
PRISMA-P: Preferred Reporting Items for Systematic Reviews and Meta-Analyses–Protocols
