# Supplementary material for: Donor Risk Factors Affecting Graft Survival in Pediatric Kidney Transplants: Protocol for a Systematic Review and Meta-Analysis
Source: JMIR Res Protoc. 2026 Feb 12;15:e71620. doi: 10.2196/71620 (PMC12900508; doi:10.2196/71620)
Supplement: Multimedia Appendix 1 [file resprot-v15-e71620-s001.docx]

Table S1. Proposed Search Terms

| **Databases** | **Search Terms** |
| --- | --- |
| MedLine | ("Pediatric"[Title/Abstract] OR "Paediatric"[Title/Abstract] OR "child*"[Title/Abstract] OR "adolescen*"[Title/Abstract] OR "teen*"[Title/Abstract] OR "Young"[Title/Abstract] OR "young adult*"[Title/Abstract] OR "Kid"[Title/Abstract] OR "Preschool"[Title/Abstract] OR "boy"[Title/Abstract] OR "girl*"[Title/Abstract] OR "male*"[Title/Abstract] OR "female*"[Title/Abstract] OR "Youth"[Title/Abstract] OR "toddler*"[Title/Abstract] OR "school child*"[Title/Abstract])  AND  ((("risk*"[Title/Abstract] OR "age"[Title/Abstract] OR "sex"[Title/Abstract] OR "gender"[Title/Abstract] OR "male*"[Title/Abstract] OR "female*"[Title/Abstract] OR "Boy"[Title/Abstract] OR "girl*"[Title/Abstract] OR "cause*"[Title/Abstract]) AND "factor"[Title/Abstract]) OR "diabet*"[Title/Abstract] OR ("hypertension"[Title/Abstract] OR "high blood pressure"[Title/Abstract] OR "HT"[Title/Abstract] OR "HTN"[Title/Abstract] OR "HBP"[Title/Abstract]) OR ("smok*"[Title/Abstract] OR "cigar*"[Title/Abstract] OR "vaping"[Title/Abstract] OR "tobacco"[Title/Abstract] OR "electronic cigar*"[Title/Abstract]) OR ("body height"[Title/Abstract] OR "height"[Title/Abstract]) OR "weight"[Title/Abstract] OR ("ethnicity"[Title/Abstract] OR "race"[Title/Abstract] OR "tribe"[Title/Abstract]) OR ((("causative"[All Fields] OR "causatively"[All Fields] OR "causatives"[All Fields] OR "cause"[All Fields] OR "caused"[All Fields] OR "causing"[All Fields] OR "etiology"[MeSH Subheading] OR "etiology"[All Fields] OR "causes"[All Fields] OR "causality"[MeSH Terms] OR "causality"[All Fields]) AND "of dead"[Title/Abstract]) OR "cause of death"[Title/Abstract]) OR ("hospitalis*"[Title/Abstract] OR "hospitaliz*"[Title/Abstract] OR "days in hospital"[Title/Abstract]) OR "CMV"[Title/Abstract] OR "HCV"[Title/Abstract] OR ("vulnerability factor*"[Title/Abstract] OR "predictor*"[Title/Abstract] OR "predisposing"[Title/Abstract] OR "contributing"[Title/Abstract] OR "donor risk factor*"[Title/Abstract])) AND ("unrelated donor*"[Title/Abstract] OR "donor selection"[Title/Abstract] OR "living donor*"[Title/Abstract] OR "tissue donor*"[Title/Abstract] OR "donor"[Title/Abstract])  AND  ("graft survival"[Title/Abstract] AND ("kidney transplant*"[Title/Abstract] OR "renal transplant*"[Title/Abstract])) |
| Scopus | TITLE-ABS-KEY ("pediatric" OR "paediatric" OR "child*" OR "adolescen*" OR "teen*" OR "young" OR "young adult*" OR "kid" OR "preschool" OR "boy" OR "girl*" OR "youth" OR "toddler*" OR "school child*")  AND  TITLE-ABS-KEY (((("risk*" OR "age" OR "sex" OR "gender" OR "male*" OR "female*" OR "boy" OR "girl*" OR "cause*") AND "factor") OR "diabet*" OR ("hypertension" OR "high blood pressure" OR "HT" OR "HTN" OR "HBP") OR ("smok*" OR "cigar*" OR "vaping" OR "tobacco" OR "electronic cigar*") OR ("body height" OR "height") OR "weight" OR ("ethnicity" OR "race" OR "tribe") OR ("cause of dead" OR "cause of death") OR ("hospitalis*" OR "hospitaliz*" OR "days in hospital") OR "CMV" OR "HCV" OR ("vulnerability factor*" OR "predictor*" OR "predisposing" OR "contributing" OR "donor risk factor*")) AND ("unrelated donor*" OR "donor selection" OR "living donor*" OR "tissue donor*" OR "donor"))  AND  TITLE-ABS-KEY ("graft survival" AND ("kidney transplant*" OR "renal transplant*"))  AND ( LIMIT-TO ( DOCTYPE , "ar" ) ) |
| CENTRAL* | (("Pediatric"[Title/Abstract/Keyword] OR "Paediatric"[Title/Abstract/Keyword] OR "child*"[Title/Abstract/Keyword] OR "adolescen*"[Title/Abstract/Keyword] OR "teen*"[Title/Abstract/Keyword] OR "Young"[Title/Abstract/Keyword] OR "young adult*"[Title/Abstract/Keyword] OR "Kid"[Title/Abstract/Keyword] OR "Preschool"[Title/Abstract/Keyword] OR "Boy*"[Title/Abstract/Keyword] OR "girl*"[Title/Abstract/Keyword] OR "male*"[Title/Abstract/Keyword] OR "female*"[Title/Abstract/Keyword] OR "Youth"[Title/Abstract/Keyword] OR "toddler*"[Title/Abstract/Keyword] OR "school child*"[Title/Abstract/Keyword])  AND  ((("risk*"[Title/Abstract/Keyword] OR "age"[Title/Abstract/Keyword] OR "sex"[Title/Abstract/Keyword] OR "gender"[Title/Abstract/Keyword] OR "male*"[Title/Abstract/Keyword] OR "female*"[Title/Abstract/Keyword] OR "boy"[Title/Abstract/Keyword] OR "girl*"[Title/Abstract/Keyword] OR "cause*"[Title/Abstract/Keyword]) AND "factor"[Title/Abstract/Keyword]) OR "diabet*"[Title/Abstract/Keyword] OR ("hypertension"[Title/Abstract/Keyword] OR "high blood pressure"[Title/Abstract/Keyword] OR "HT"[Title/Abstract/Keyword] OR "HTN"[Title/Abstract/Keyword] OR "HBP"[Title/Abstract/Keyword]) OR ("smok*"[Title/Abstract/Keyword] OR "cigar*"[Title/Abstract/Keyword] OR "vaping"[Title/Abstract/Keyword] OR "tobacco"[Title/Abstract/Keyword] OR "electronic cigar*"[Title/Abstract/Keyword]) OR ("body height"[Title/Abstract/Keyword] OR "height"[Title/Abstract/Keyword]) OR "weight"[Title/Abstract/Keyword] OR ("ethnicity"[Title/Abstract/Keyword] OR "race"[Title/Abstract/Keyword] OR "tribe"[Title/Abstract/Keyword]) OR ("cause of dead"[Title/Abstract/Keyword] OR "cause of death"[Title/Abstract/Keyword]) OR ("hospitalis*"[Title/Abstract/Keyword] OR "hospitaliz*"[Title/Abstract/Keyword] OR "days in hospital"[Title/Abstract/Keyword]) OR "CMV"[Title/Abstract/Keyword] OR "HCV"[Title/Abstract/Keyword] OR ("vulnerability factor*"[Title/Abstract/Keyword] OR "predictor*"[Title/Abstract/Keyword] OR "predisposing"[Title/Abstract/Keyword] OR "contributing"[Title/Abstract/Keyword] OR "donor risk factor*"[Title/Abstract/Keyword])) AND ("unrelated donor*"[Title/Abstract/Keyword] OR "donor selection"[Title/Abstract/Keyword] OR "living donor*"[Title/Abstract/Keyword] OR "tissue donor*"[Title/Abstract/Keyword] OR "donor"[Title/Abstract/Keyword])  AND  ("graft survival"[Title/Abstract/Keyword]) AND ("kidney transplant*"[Title/Abstract/Keyword] OR "renal transplant*"[Title/Abstract/Keyword]) |
| Web of Science | **AB=("pediatric" OR "paediatric" OR "child*" OR "adolescen*" OR "teen*" OR "young" OR "young adult*" OR "kid" OR "preschool" OR "boy" OR "girl*" OR "youth" OR "toddler*" OR "school child*")**  AND  **AB=(((("risk*" OR "age" OR "sex" OR "gender" OR "male*" OR "female*" OR "boy" OR "girl*" OR "cause*") AND "factor") OR "diabet*" OR ("hypertension" OR "high blood pressure" OR "HT" OR "HTN" OR "HBP") OR ("smok*" OR "cigar*" OR "vaping" OR "tobacco" OR "electronic cigar*") OR ("body height" OR "height") OR "weight" OR ("ethnicity" OR "race" OR "tribe") OR ("cause of dead" OR "cause of death") OR ("hospitalis*" OR "hospitaliz*" OR "days in hospital") OR "CMV" OR "HCV" OR ("vulnerability factor*" OR "predictor*" OR "predisposing" OR "contributing" OR "donor risk factor*")) AND ("unrelated donor*" OR "donor selection" OR "living donor*" OR "tissue donor*" OR "donor"))**  AND  **AB=("graft survival" AND ("kidney transplant*" OR "renal transplant*"))** |
| EMBASE | (("pediatric*" or "child*" or "adolescen*" or "teen*" or young or "young adult*" or kid or preschool or boy or girl or youth or toddler or "school child*") and (((("risk*" or age or sex or gender or male or female or boy or girl or cause) and factor) or ("diabet*" or hypertension or high blood pressure or HTN or HT or HBP) or ("smok*" or cigar or vaping or tobacco or "electronic cigar*") or body height or height or weight or ethnicity or race or tribe or cause of dead or cause of death or "hospitalis*" or "hospitaliz*" or days in hospital or CMV or HCV or "vulnerability factor*" or predictor or predisposing or contributing or donor risk factor) and (unrelated donor or donor selection or living donor or tissue donor or donor)) and (graft survival and ("kidney transplant*" or "renal transplant*"))).ab.  AND  “Article”[Publication Type] |

*CENTRAL: Cochrane Central Register of Controlled Trials
